# Multimodal Radiomic Features for the Predicting Gleason Score of Prostate Cancer

**DOI:** 10.3390/cancers10080249

**Published:** 2018-07-28

**Authors:** Ahmad Chaddad, Michael J Kucharczyk, Tamim Niazi

**Affiliations:** 1 Division of Radiation Oncology, Department of Oncology, McGill University, Montreal, QC H3S 1Y9, Canada; michael.kucharczyk@medportal.ca (M.J.K.); tniazi@jgh.mcgill.ca (T.N.); 2 The Laboratory for Imagery, Vision and Artificial Intelligence, École de Technologie Supérieure, Montreal, QC H3C 1K3, Canada

**Keywords:** biomarkers, Gleason score, radiomics, prostate cancer

## Abstract

Background: Novel radiomic features are enabling the extraction of biological data from routine sequences of MRI images. This study’s purpose was to establish a new model, based on the joint intensity matrix (JIM), to predict the Gleason score (GS) of prostate cancer (PCa) patients. Methods: A retrospective dataset comprised of the diagnostic imaging data of 99 PCa patients was used, extracted from The Cancer Imaging Archive’s (TCIA) T2-Weighted (T2-WI) and apparent diffusion coefficient (ADC) images. Radiomic features derived from JIM and the grey level co-occurrence matrix (GLCM) were extracted from the reported tumor locations. The Kruskal-Wallis test and Spearman’s rank correlation identified features related to the GS. The Random Forest classifier model was implemented to identify the best performing signature of JIM and GLCM radiomic features to predict for GS. Results: Five JIM-derived features: contrast, homogeneity, difference variance, dissimilarity, and inverse difference were independent predictors of GS (*p* < 0.05). Combined JIM and GLCM analysis provided the best performing area-under-the-curve, with values of 78.40% for GS ≤ 6, 82.35% for GS = 3 + 4, and 64.76% for GS ≥ 4 + 3. Conclusion: This retrospective study produced a novel predictive model for GS by the incorporation of JIM data from standard diagnostic MRI images.

## 1. Introduction

In both Canada and the United States, prostate cancer (PCa) is the most common non-dermatological cancer in men [1,2]. In 2017 in Canada, there were 21,300 men diagnosed with PCa, and among them 4100 related deaths [3]. Screening for PCa has been investigated in numerous randomized control trials with various caveats. Even with low morbidity rates [4], the questionable net benefit from screening has resulted in guidelines that emphasized the need to increase our pre-test probability with non-invasive approaches prior to prostate biopsy.

Generally, patients with PCa are classified into either a low, intermediate, or high risk group based on their prostate-specific Antigen (PSA) level, pathological assessment/Gleason Score (GS) [5,6], and clinical stage (i.e., T stage) [7]. PSA is not an accurate biomarker alone, providing a false-positive result for PCa in up to 50% of men [8,9]. Currently, management decisions are heavily based on the histopathological tumor-grading assessment from trans-rectal ultrasound guided core-needle biopsies. Even with improvements in biopsy localization, the biopsies only provide a limited geographical sampling of the tumour. Understandably, biopsy-derived GS has been shown to significantly differ from the final GS following radical prostatectomy [10]. Complicating assessment further, there is significant heterogeneity and dependence on expertise to accurately determine GS from biopsies [11,12,13]. A non-invasive biomarker to predict the GS would thus have value in selecting patients for biopsy and for assisting with the scrutinization of discordant biopsy results relative to clinical findings.

Several studies show the potential of multiparametric magnetic resonance imaging (mpMRI) for predicting the GS [14,15,16]. Specifically, a modern reporting system (i.e., Prostate Imaging Reporting and Data System, known as PI-RADS) is designed to improve focal lesion detection, localization, characterization, and risk stratification in patients with suspected cancer. It accomplishes this through explicit technical recommendations for MRI acquisition and a scoring system for image interpretation [17]. Updates to PI-RADS have reduced, but by no means eliminated, inter-reader variability. There is an acknowledged need to improve lesion characterization in future iterations of the tool [16]. The field has further diversified with studies using less conventional imaging features to correlate to the GS [18,19,20,21]. While these studies used uni- and mpMRI images, there is limited work that implements a more novel approach—encoding the cross structure/texture of a mpMRI.

The most recent studies of non-invasive MRI-based techniques have implemented radiomics analysis to further increase our ability to prognosticate PCa [18,21,22,23,24]. Radiomics analysis is an approach that incorporates various radiological features, namely texture and shape characteristics/features, to predict biological characteristics. An increasingly implemented radiomic feature is gray level co-occurrence matrices (GLCM) [25]. GLCM encodes the texture of a region of interest (ROI) and measures the heterogeneity within it. Clinically, the ROI is typically suspicious for malignancy. At a technical level, these texture features are computed from each of MRI sequence then used as an input of the classifier model to predict for or prognosticate a PCa [23].

In this retrospective study, we derived a novel radiomic model based on joint intensity matrices (JIMs), then evaluated this model’s ability predict the GS in PCa patients. These imaging characteristics effectively encode the joint co-occurrence of image intensity values occurring within the tumor on mpMRI sequences. We hypothesized that these joint distributions of intensities would predict for cellular heterogeneity within the biopsy confirmed PCa, which are related to the GS. To our knowledge, this is the first study that has implemented cross-modality intensity statistics for identifying radiomic features associated with GS. Specifically, we investigated the capacity of different JIM features, derived from two different MRI sequences, to predict a PCa’s GS in either the transitional or peripheral zone.

## 2. Materials and Methods 

**Figure 1:**
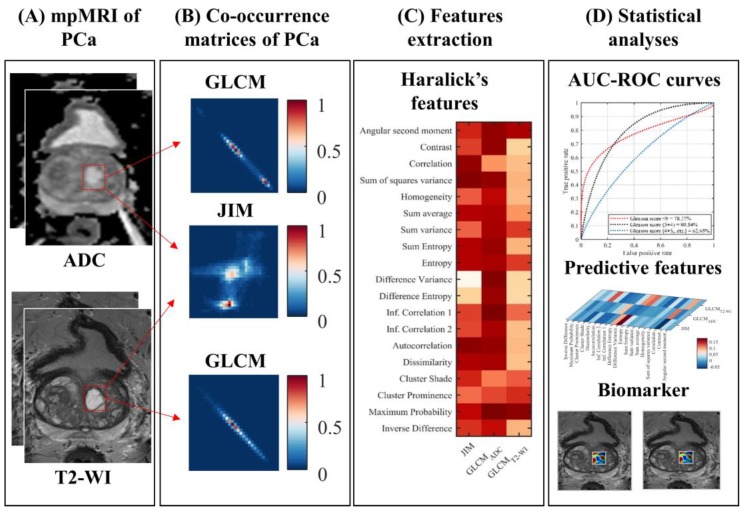
Schema of the utilized methodology for a GS prediction model for prostate cancer. (**A**) T2-WI and ADC MRI images of 99 patients with biopsy-confirmed and MRI-localized PCa are extracted; (**B**) Co-occurrence matrices (i.e., JIM and GLCMs) were computed from ROI’s which corresponded to biopsy-proven sites of PCa; (**C**) The 19 features encoded within the GLCMs and JIM are extracted; (**D**) Statistical analyses performed uni- and multivariate analyses of these features to generate a model predictive of the GS.

This subsection and Figure 1 summarize our methods, and then greater detail is expanded upon in the following subsections. Our study utilized an automated cross-modality intensity statistic to identify the biomarker features associated with GS in PCa patients. First, the ADC and T2-WI MRI images of 99 PCa patients were accessed. PCa regions were identified based on the published centroid/location of PCa nodules (i.e., the ROI) on a publicly accessible database [26]. GLCMs and the JIM were then computed for each ROI. Nineteen different quantifier functions were then encoded into the co-occurrence matrices. Analyses were conducted to assess the usefulness of these features to predict the GS. In a first analysis, a Kruskal-Wallis test and Spearman’s correlation coefficient are used to find features associated with the three GS groupings—G1 when GS ≤ 6; G2 when GS = 3 + 4; and G3 when GS ≥ 4 + 3. Then, all texture features were used to train a random forest (RF) model to predict for a GS grouping. The RF model identified features with a high predictive value for discriminating between GS groups in this population of PCa nodules.

### 2.1. Data Description

We retrospectively reviewed the 99 PCa patients of the SPIE-AAPM-NCI Prostate MR Gleason Grade Group Challenge (http://spiechallenges.cloudapp.net/competitions/7) and the Cancer Imaging Archive (TCIA), a publicly available medical image repository. PCa patients have been previously de-identified by SPIE-AAPM-NCI and TCGA/TCIA. As such, no institutional review board or Health Insurance Portability and Accountability Act approval was required for this study. 

As explained by the TCIA [26], each MRI series was read and reported by a radiologist with expertise in prostate MRI. Areas considered significantly suspicious for malignancy were biopsied with MRI-confirmed tumor localization with the biopsy needle in-situ. Biopsy specimens were graded by an expert genitourinary pathologist and thus achieved the gold-standard for pre-prostatectomy pathological assessment.

Images were acquired by either a Siemens 3T MAGNETOM Trio or Skyra MRI. Pixel spacing, slice thickness and contrast varied within the included cohort. These differences were overcome by all images being resampled to a common voxel resolution of 1 mm^3^, for a total size of 320 × 320 × 19 voxels. Intensities within each volume were normalized to the [0,1] range. The ROI of 21 × 21 × 10 voxels was localized from the predefined centroid coordinates provided by the SPIE-AAPM-NCI Prostate MR Gleason Grade Group Challenge and then extracted from the T2-WI and ADC images. Radiomic features were extracted from the computed co-occurrence matrices (i.e., GLCM and JIM) of the 3D ROI.

### 2.2. Proposed Radiomic Features

Conventional GLCM-based features are a technique that characterizes the texture of an image but is unable to evaluate the texture across multiple MRI sequences. To overcome this limitation for this study’s mpMRI images (i.e., T2-WI and DWI), we proposed a joint intensity matrix (JIM)-based model instead. Elaborated upon in the existing literature [27], GLCM estimated the joint probability of observing a pair of intensity values in two ROI voxels whose relative position is defined by a distance *d* and angle *θ*. Therefore, JIM computes the joint intensity distribution between 3D images of different modalities, given two images derived from two modalities *I*_1_ (i.e., T2-WI) and *I*_2_ (i.e., DWI), with segmented 3D tumor region *R* and *i* and *j* denoted as intensity values from the set {1, …, N}. The JIM corresponding to distance *d* and angle *θ* can be obtained as:

(1) $$JIM_{d,\theta }\left(i,j\right)=\;\sum_{x\in R}\{\begin{array}{c} 1,\;\mathrm{if}\;I_{1}\left(x\right)=i\;\mathrm{and}\;I_{2}\left(L_{d,\theta }\left(x\right)\right)=j\\ 0,\;\mathrm{otherwise}\; \end{array}$$

in which $L_{d,\theta }\left(x\right)$ corresponds to the voxel located at distance *d* and angle *θ* from ***x***. Thus, both JIMs and GLCMs have 2D outputs computed from 3D datasets. To obtain probability distributions, JIMs were then unit normalized:

(2) $${\hat{JIM}}_{d,\theta }\left(i,j\right)=\frac{J_{d,\theta }\left(i,j\right)}{\sum_{i,j=1}^{N}J_{d,\theta }\left(i,j\right)}$$

Note that the JIM is a square matrix. In this study, we considered five grey levels (N) in which N = {8, 16, 32, 64, 128}. Thus, JIM size was N × N (i.e., 8 × 8, 16 × 16, 32 × 32, 64 × 64, 128 × 128).

The GLCM can be conceptualized as a special case of JIM in which the same image is used for I_1_ and I_2_. Thus, the advantage of JIM is that it considers the cross-modality dependencies between voxel intensities, in addition to their spatial relationship. Figure 1 shows an example of two MRI modalities, T2-WI and ADC, with JIM and GLCM matrices computed from a 3D ROI extracted from a confirmed PCa. More information is available in a JIM image, as opposed to the GLCM images, as illustrated in Figure 1 (Co-occurrence matrices of PCa).

To characterize the texture derived from a PCa, we quantified the 2D matrices of JIM and GLCM with the 19 functions previously proposed by Haralick’s (i.e., angular second moment, contrast, correlation, sum of squares variance, homogeneity, sum average, sum variance, sum entropy, entropy, difference variance, difference entropy, information correlation_1_, information correlation_2_, autocorrelation, dissimilarity, cluster shade, cluster prominence, maximum probability, and inverse difference) [28]. Individual values are reported in Supplementary materials Table S1. Each texture feature is represented by the average of feature values corresponding to five quantization levels (8, 16, 32, 64, and 128), four direction/orientations (0°, 45°, 90°, and 135°), and four distances (*d* = 1, 2, 3, and 4 voxels). Using this strategy, each PCa tumor was represented by a set of 57 features—19 functions computed from two GLCMs (i.e., T2-WI and ADC) and a third from a JIM (i.e., T2-WI + ADC) specific to a given tumor’s ROI.

### 2.3. Statistical Analysis

Several analyses were performed to identify relevant features that predicted the GS grouping of each PCa nodule. We first used Spearman’s rank correlation [29] to compute the correlation value between each of texture features and the GS group for the corresponding PCa patients. We then applied the Kruskal-Wallis test to compare the distribution of feature values across GS groups (i.e., G1 when GS ≤ 6; G2 when GS = 3 + 4; G3 when GS ≥ 4 + 3). To account for our two models, which each incorporated 57 comparisons (19 quantifiers functions × 3 co-occurrence matrices—GLCM or JIM), the Holm-Bonferroni procedure assessed for statistical significance at *p* < 0.05. This correction technique has been established to be more powerful for similar datasets compared to the standard Bonferroni method [30].

To illustrate the impact of JIM features, relative to the GLCMs’, we input these features into a random forest (RF) classifier. Five hundred random trees were utilized to predict the GS groupings of the PCa tumors. To show the area under the curve (AUC) for the ROC, we considered the RF as a binary classification problem: G1 vs. all, G2 vs. all, and G3 vs. all. Note that multiple classifier models could be used for the prediction; we chose the RF as an accepted model that performs well when the number of training samples is limited. This is secondary to RF employing a bagging strategy that reduces errors due to sample variance. Additionally, RF reliably allowed for the inspection of features to predict GS group [31].

For this analysis, a balance between two classes was considered. For example, to predict the G1 (i.e., GS ≤ 6), 60 PCa patients were randomly assigned to either a training (i.e., *n* = 20 G1 and 20 G2 + G3) or testing (*n* = 10 G1 and 10 G2 + G3) dataset. We then computed ROC’s AUC to classify G1 from the G2 and G3. The same approach (i.e., one vs. all) was repeated to compute the AUC for G2 versus G3.

To identify the predictive features, we used the texture features and measured the increase in RF error resulting from the permutation of feature values across out-of-bag observations. The importance value of features was computed for every RF tree then averaged over the entire ensemble. These values were then normalized by dividing them by the ensemble’s standard deviation. A positive importance value indicated that the feature was predictive, whereas negative importance values suggested that the feature had no predictive value [31].

## 3. Results

### 3.1. Patient Characteristics

We considered a dataset of 99 PCa patients collected from the SPIE-AAPM-NCI and the Cancer Imaging Archive (TCIA). The patient characteristics are reported in Table 1. There was a relatively even distribution between G1 (*n* = 30), G2 (*n* = 39), and G3 (*n* = 30).

### 3.2. Differences and Correlation Analysis

**Figure 2:**
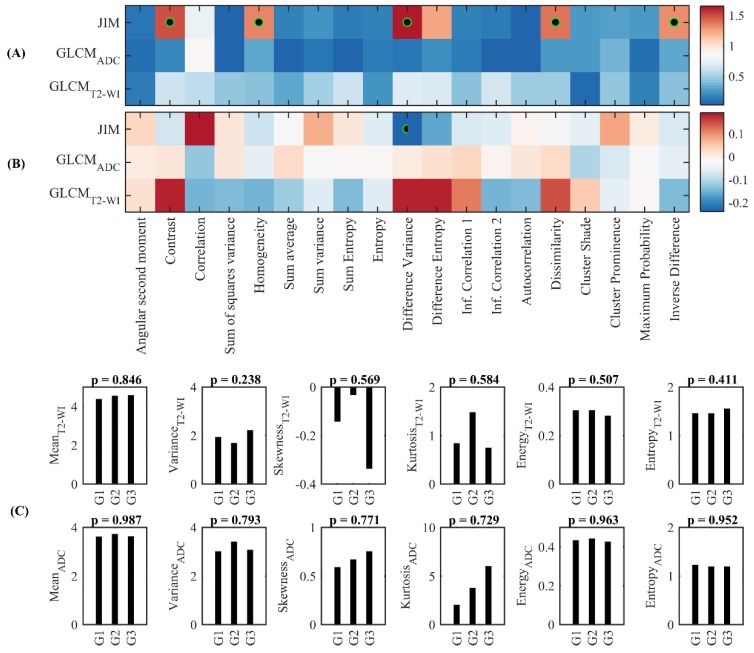
Univariate prediction of GS using texture features (**A**) Kruskal-Wallis significance test compared the three GS groupings. (**B**) Spearman’s rank correlation coefficient between texture features and the GS groupings. Significant features (*p* < 0.05) are indicated with a black-green circle. (**C**) Average of the histogram features derived from T2-WI (i.e., first row) and ADC (i.e., second row) image value (Histogram features: Mean, Variance, Skewness, Kurtosis, Energy, and Entropy) across the three groups of GS (i.e., G1, G2, and G3).

Figure 2A shows the heatmap of *p*-values, in a negative log_10_ scale, obtained by the Kruskal-Wallis test comparing the feature value distributions of PCa tumors’ GS groupings. Five features derived from JIM were significant with corrected *p* < 0.05: contrast, homogeneity, difference variance, dissimilarity, and inverse difference (Table 2). The difference variance feature reported the highest differences between GS groupings.

Spearman’s rank correlation between texture features and GS groupings showed a low correlation value and a maximum absolute value of 0.23. The difference variance feature, derived from the JIM, showed a negative correlation value of 0.23 with a corrected *p* < 0.05 (Figure 2B).

Figure 2C illustrates the average value of the standard histogram features (Mean, Variance, Skewness, Kurtosis, Energy, and Entropy) computed from the series derived from the ADC and T2-WI image values of PCa patients. We observed that the average of the skewness and kurtosis features derived from ADC images increased with the GS, while entropy was inversely related to the GS. None of these features demonstrated statistical significance via the Wilcoxon test (*p* < 0.05).

### 3.3. Gleason Score Prediction

**Figure 3:**
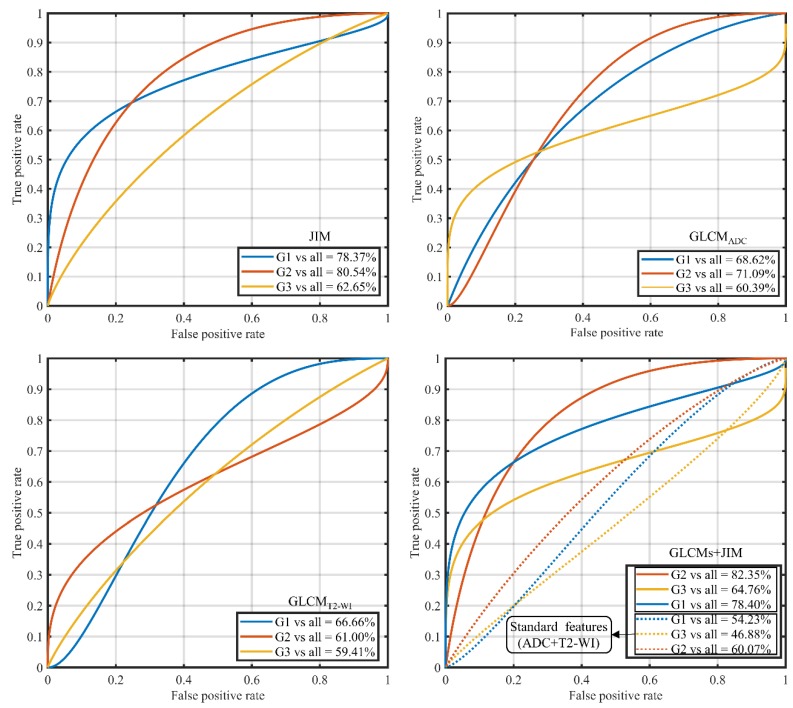
Multivariate analysis of texture features and RF classifier model. The AUC for predicting G1 (GS ≤ 6), G2 (GS = 3+4), and G3 (GS ≥ 4 + 3) using the JIM (19 features), GLCM_ADC_ (19 features), GLCM_T2-WI_ (19 features), all combined features (GLCMs + JIM, 19 × 3 features), and all of the standard features (histogram features derived from ADC and T2-WI images of PCa, 6 × 2 features). For training (*n* = 40 samples) and testing (*n* = 20 samples), balance samples were considered.

In the multivariate prediction analysis based on the RF classifier, for all GS groupings, we found that the AUC values using all the JIM features were higher than those based on all of the GLCM features (Figure 3). Note that the RF classifier models should be able to selected the relevant features in automatic fashion [32,33]. As previously mentioned, balance samples were utilized for the training/model development (*n* = 40) and testing/validation (*n* = 20) for each of the GS groups. 

In Figure 3, AUCs based on JIM features had a value of 78.37% for G1, 80.54% for G2, and 62.65% for G3, while the conventional GLCM features had AUC values of 68.62%, 71.09%, and 60.39%. For the GLCM features derived from ADC and T2-WI, the AUC values were 66.66%, 61.00%, and 59.41% for G1, G2, and G3, respectively.

However, combined features (GLCMs + JIM) had the highest AUC values of 78.40%, 82.35%, and 64.76% for G1, G2, and G3, respectively. The combined features led to the highest prediction score among all the GS groupings. Generally, AUC values from RF models are consistent with results of the Kruskal-Wallis test and Spearman correlation. In this dataset, GS groupings predicted by JIM features were of the greatest Kruskal-Wallis test significance and Spearman correlation value.

The standard features (Mean, Variance, Skewness, Kurtosis, Energy, and Entropy) were also derived from the PCa ROIs on T2-WI and ADC series. This produced AUC values of 54.23%, 60.07%, and 46.88% for G1, G2, and G3, respectively, lower than those obtained using the GLCMs and/or JIM features.

### 3.4. Predictive Feature Analysis

**Figure 4:**
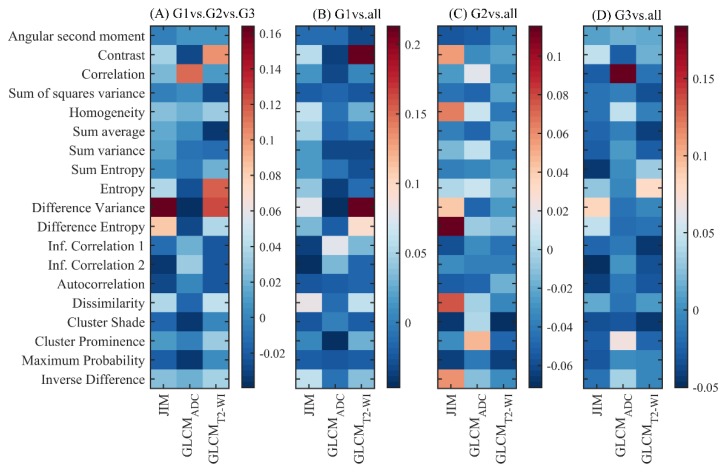
Importance of individual features for predicting GS groups using the RF classifier. Positive and negative values correspond respectively to predictive and non-predictive features.

The predictive feature of GS, as measured by the RF classifier, is shown in Figure 4. We found several GLCM and JIM features with predictive value (i.e., importance score > 0). To predict the tumors with a GS ≤ 6 (Figure 4B), we found that the difference variance and contrast derived from GLCM of T2-WI were the most discriminative features. Figure 4C shows that the JIM features difference entropy and dissimilarity were the most predictive feature for G2 (GS = 3 + 4), while correlation derived from the GLCM of the ADC images was the most predictive feature to predict the G3 (GS = 4 + 3 and GS > 7).

The difference variance feature derived from JIM had the highest importance value for classifying between G1, G2, and G3 (Figure 4A). Difference variance-derived JIM was also previously found to be significant in the Kruskal-Wallis and Spearman correlation analysis (Figure 2). Inconsistencies between the Kruskal-Wallis significance of other features and RF importance could be explained by the randomness of feature selection in RF, in which discriminative features can be ignored in favor of more predictive ones.

## 4. Discussion

Radiomic analysis has increasingly demonstrated the potential to translate the intrinsic heterogeneity of a tumor’s MRI images into a radiomic signature or biomarker. In this context, a constellation of texture features (i.e., the aforementioned radiomic signature) offers increasing potential to accelerate precision medicine by exploring the relationships between tumor image heterogeneity, genomic heterogeneity, treatment resistance, and metastatic probability [23,34,35,36,37,38,39,40]. Texture features derived from GLCMs are the most popular technique with which to measure image heterogeneity [28]. Intrinsic to GLCM use is the extraction of features from one set of imaging parameters, independent of the data embedded elsewhere in the data set. Unfortunately, this has prevented the full use of information at hand in the conventional MRI assessment of PCa tumors—there is a wealth of encoding relationships to be investigated.

In this study, the multimodal texture features relationships with PCa tumors are represented using a JIM—a computation that translates image heterogeneity into texture predictors. The JIM approach was applied to MRI images of PCa tumors to establish features associated with the GS. This approach had advantages over the traditional assessments, because it provides insight into the relationship between intensity values across multi-parametric images. Our results showed five different JIM-derived features (contrast, homogeneity, difference variance, dissimilarity, and inverse difference) that have the capacity to compare between GS groups with a corrected *p* < 0.05. In contrast, the median of ADC images and the classical GLCM texture features were unable to discriminate between the GS groups (Figure 2).

Predictive features of the RF classifier model demonstrated that the difference variance feature derived from JIM was the most predictive feature of GS. Difference variance encoded the texture and described the variation between the texture (i.e., heterogeneity).

Our study is consistent with most of the other literature that has implemented texture analysis to analyze PCa. For example, entropy has been demonstrated to be a good biomarker with GS [23]. Combined features derived from T2-WI and magnetic resonance spectroscopy images were able to classify between low and high GS [21]. Average ADC image/maps has also been demonstrated to be a biomarker for GS [18]. Additionally, the majority of the prior studies have been focused on classifying cancerous regions from benign structures [41,42,43,44].

There are fewer works that seek to determine a potential tumor’s GS. Higher GS cancers have been found to be associated with relatively high ADC entropy and low ADC energy, in comparison with low GS cancers [23]. Texture features have been used to classify between GS = 6 vs. GS ≥ 7 and 7(3 + 4) vs. 7(4 + 3) with reasonable accuracy. However, this approach utilized the imputation to overcome the unbalanced samples [45]. For the volume feature derived from mpMRI, the Spearman correlation coefficient has shown a moderate correlation with GS [46]. However, volume as a feature is based on the manual segmentation. Manual segmentation can suffer from inter-reader variability and thus this result could be contaminated with a high bias. For these reasons, our study only implemented texture analysis. We demonstrated the mpMRI derived joint texture’s feasibility as a potential technique to predict a PCa tumour’s GS—the strongest biological correlate of a PCa tumor’s aggressiveness.

Our study has limitations intrinsic to a retrospective analysis of a publicly available data set. First, the study was a retrospective analysis of a relatively small group of patients (*n* = 99). Thus, the results require prospective validation on a larger scale prior to broader clinical application. Second, our work did not explore other pertinent tumor features that could introduce bias secondary to manual segmentation. These features included: volume, solidity, convexity, and eccentricity of a lesion on MRI. Thirdly, our use of a publicly available database increased the transparency of our work but limited the ability to evaluate ancillary clinical data. Thus, other pertinent features, namely, a patient’s PSA and MRI PI-RADS score, could not be incorporated into our model. These clinical characteristics are a fundamental component of the clinician’s assessment and must be integrated into the next iteration of this technique. As the next step to building upon the works herein, we foresee such features and clinical characteristics incorporated into convolution neural networks and then applied to a variety of MRI sequences in a prospective setting.

## 5. Conclusions

This study presented a novel type of radiomic analysis. It implemented features based on JIM, which better describes the heterogeneity across mpMRI images. Our study emphasized that JIM features could be complementary to GLCM techniques for the prediction of the GS in PCa patients. Results demonstrated the advantages of JIM over standard GLCM features and suggest that the difference variance derived JIMs could be used as a biomarker for predicting GS in PCa tumors.

## Figures and Tables

**Table 1:** Patients characteristics related to Gleason score.

Gleason (n)	Gleason Score	Our Classification (n)
Grade Group 1 (30)	≤6	G1 (30)
Grade Group 2 (39)	3 + 4 = 7	G2 (39)
Grade Group 3 (17)	4 + 3 = 7	G3 (30)
Grade Group 4 (7)	4 + 4 = 8; 3 + 5 = 8; 5 + 3 = 8	
Grade Group 5 (6)	9 or 10	

**Table 2:** Summary of five significant JIM features (Median-IQR) between Gleason score (GS) groups.

JIM Features	GS ≤ 6, *n* = 30	GS = 3 + 4, *n* = 39	GS ≥ 3 + 4, *n* = 30	*p*-Value
Contrast	536.5119–325.99	735.9059–492.77	438.9053–373.11	0.03
Homogeneity	0.1365–0.06	0.1117–0.08	0.1362–0.07	0.04
Difference variance	159.4869–49.76	188.8752–111.58	129.6384–65.47	0.02
Dissimilarity	14.5873–4.68	17.1876–7.19	13.6761–6.77	0.04
Inverse difference	0.2093–0.06	0.1827–0.08	0.2091–0.08	0.04

## Supplementary Materials

The following are available online at http://www.mdpi.com/2072-6694/10/8/249/s1, Table S1: Description of features extracted from JIM/GLCM of PCa tumors.
