# Supplementary material for: Multimodal Radiomic Features for the Predicting Gleason Score of Prostate Cancer
Source: Cancers (Basel). 2018 Jul 28;10(8):249. doi: 10.3390/cancers10080249 (PMC6116195; doi:10.3390/cancers10080249)
Supplement: Supplementary file 1 [file cancers-10-00249-s001.pdf]

# Supplementary materials: Multimodal Radiomic Features for the Predicting Gleason Score of Prostate Cancer

**Ahmad Chaddad, Michael J Kucharczyk and Tamim Niazi**

**Table S1. Description of features extracted from JIM/GLCM of PCa tumors.**

| Features/functions | Description |
|---|---|
| **Angular second moment** | Measures the textural uniformity and detects disorders in textures |
| **Contrast** | Measures the local intensity variation |
| **Correlation** | Describes the linear dependency of gray level values relative to their respective intensity of the JIM/GLCM |
| **Sum of squares variance** | Measures the distribution of neighboring intensity level pairs compared to the average of intensity level of the JIM/GLCM |
| **Homogeneity** | Measures the similarity of the distribution of elements of the JIM/GLCM relative to the JIM/GLCM diagonal |
| **Sum-average** | Measures the relationship between occurrences of pairs with lower intensity values and occurrences of pairs with higher intensity values |
| **Sum-variance** | Describes the extent of variation of elements which differ from the average value of the JIM/GLCM. |
| **Sum-entropy** | Representation the sum of the differences in neighborhood intensity values. |
| **Entropy** | Describes the randomness of the JIM/GLCM |
| **Difference variance** | Measure of heterogeneity which emphasizes intensity level pairs which deviate more from the mean. |
| **Difference entropy** | Measure of the randomness/variability in the differences of neighborhood intensity values. |
| **Information of correlation 1** | Measure of the differences of randomness (entropy) |
| **Information of correlation 2** | Measure of the differences of randomness using exponential formula |
| **Autocorrelation** | Measure of the magnitude of the fineness and coarseness of texture |
| **Dissimilarity** | Describes the contrast of the local region |
| **Cluster shade** | Measure of the skewness and uniformity of the JIM/GLCM |
| **Cluster prominence** | Measure of the skewness and asymmetry of the JIM/GLCM |
| **Maximum probability** | Representation of the occurrences of the most predominant pair of neighboring intensity values. |
| **Inverse difference** | Measure of the local homogeneity of an image |
